# *Staphylococcus saprophyticus* Recovered from Humans, Food, and Recreational Waters in Rio de Janeiro, Brazil

**DOI:** 10.1155/2017/4287547

**Published:** 2017-05-24

**Authors:** Viviane Santos de Sousa, Ana Paula de Souza da-Silva, Leif Sorenson, Raphael Paiva Paschoal, Renata Fernandes Rabello, Eloiza Helena Campana, Márcia Soares Pinheiro, Lyssa Oliveira Ferreira dos Santos, Natacha Martins, Ana Carolina Nunes Botelho, Renata Cristina Picão, Sérgio Eduardo Longo Fracalanzza, Lee Woodland Riley, George Sensabaugh, Beatriz Meurer Moreira

**Affiliations:** ^1^Laboratório de Investigação em Microbiologia Médica, Universidade Federal do Rio de Janeiro, Rio de Janeiro, RJ, Brazil; ^2^School of Public Health, University of California, Berkeley, Berkeley, CA, USA; ^3^Instituto Biomédico, Universidade Federal Fluminense, Niterói, RJ, Brazil

## Abstract

*Staphylococcus saprophyticus* is an important agent of urinary tract infection (UTI) in young women, but information about this pathogen in human microbiota and in common environment is lacking. The aim of this study was to characterize* S. saprophyticus* isolates from genitoanal microbiota of 621 pregnant women, 10 minas cheese packs, and five beaches in Rio de Janeiro city and compare PFGE profiles of these isolates with five UTI PFGE clusters described in this city. We investigated 65* S. saprophyticus* isolates from microbiota, 13 from minas cheese, and 30 from beaches and 32 UTI isolates. Antimicrobial resistance was determined by disk diffusion, MIC by agar dilution, and PCR. Erythromycin-resistance genes* erm*(C),* msr*(A),* msr*(B),* mph*(C), and* lin*(A) were found in 93% of isolates. Trimethoprim-sulfamethoxazole resistance correlated with* dfrG* or* dfrA* genes. Three cefoxitin-resistant isolates carried the* mecA* gene. All isolates obtained from cheese were susceptible to all antimicrobial agents. Six of 10 pregnant women with >1 isolate had monoclonal colonization. Isolates from pregnant women shared 100% similarity with UTI PFGE cluster types A and E obtained almost 10 years previously, suggesting temporal persistence of* S. saprophyticus*. Antimicrobial resistance of beach isolates reflected the profiles of human isolates. Taken together, results indicate a shared source for human and environmental isolates.

## 1. Introduction

The source or reservoir of* Staphylococcus saprophyticus* for humans is not fully known. This coagulase-negative microorganism is recognized to cause urinary tract infection (UTI) in sexually active young women [1]. Despite the numerous reports of this microorganism in food [2–4], the relationship between these findings and the occurrence of UTI in humans has not been demonstrated [5]. In Brazil,* S. saprophyticus* was described in minas cheese [6], one of the most popular kinds of fresh cheese in the country, and in the water of a polluted river [7]. In addition, reports of* S. saprophyticus* in the marine environment [8] and food derived from fish [9, 10] draw attention to the spread of this microorganism. These may indicate that nonhuman sources of* S. saprophyticus* colonization may include, in addition to food, contact with the marine environment, an unexplored phenomenon.

In a previous report, we described the distribution of pulsed-field gel electrophoresis (PFGE) types in a population of 32* S. saprophyticus* isolates from community-acquired UTI in Rio de Janeiro from 2005 to 2006 [11]. Clusters of isolates with indistinguishable PFGE patterns were observed among unrelated individuals, indicating possible point sources of this uropathogen. Such putative sources were not investigated in the previous study, and much of the epidemiology of* S. saprophyticus* UTI is yet to be described.

The emergence of antimicrobial resistance in uropathogens is a worldwide concern. The characterization of resistance determinants in UTI pathogens is important for the clinical management of UTI. Although* S. saprophyticus* may carry many resistance genes, most studies include this organism in a coagulase-negative group or deal with low numbers of samples [12–17], and the resistance determinants in clinical isolates are rarely described. Reports of the presence in the urogenital microbiota associated with UTI by this microorganism date from the 1980s [18]; therefore, recent evidence of this colonization in the human microbiota is lacking.

The aim of the present study was to characterize antimicrobial resistance genes in* S. saprophyticus* isolates from UTI cases, the microbiota of pregnant women, and two potential colonization sources: artisanal minas cheese and the waters of five beaches in the city of Rio de Janeiro. In addition, we aimed to compare the PFGE band profiles of these isolates with five uropathogenic* S. saprophyticus* clusters previously described in this city [11].

## 2. Materials and Methods

### 2.1. Bacterial Isolation and Identification

**Figure 1:**
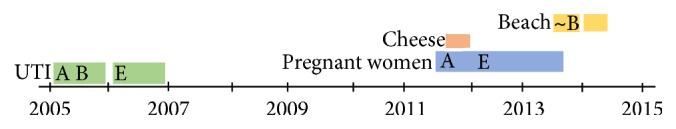
Distribution of isolates obtained from different sources in Rio de Janeiro according to time. Capital letters represent the urinary tract infection (UTI) PFGE types. “~B”: beach isolate with 82% similarity with UTI type B.

We obtained isolates from four sources: UTI [11], pregnant women's microbiota, minas cheese, and beach waters. We considered as suspect of* S. saprophyticus* the colonies from cultures of cheese and the microbiota samples that grew on Mannitol Salt Agar (BD, New Jersey, USA) with 100 *µ*g/mL of novobiocin (Inlab, São Paulo, Brazil) and the colonies from beach water samples that grew on the same selective medium with addition of 0.005% sodium azide [28]. The distribution of isolates according to time (years) and source of detection is shown in Figure 1.

Isolates from UTI were obtained from 32 patients of two walk-in clinics in Rio de Janeiro city during March–November 2005 and March–November 2006 [11]. PFGE typing revealed the presence of five time-based clusters (types A–E) in isolates of patients coming from places spread in the community. One isolate of each cluster type was used in comparisons with PFGE types obtained in the present study. Isolates from pregnant women's microbiota were obtained from vaginal and anal swab specimens stored during surveillance for* Streptococcus agalactiae* colonization. Samples were collected from 621 healthy pregnant women in the 35–37th weeks of gestation, from August/2011 to August/2013; five colonies from vaginal and anal sites of each patient were selected for* S. saprophyticus* screening. Ten minas cheese packs from three different brands were obtained in November 2011 from different stores in eight neighborhoods in Rio de Janeiro city, taken to the lab in original packaging, and coded with roman numbers. Analysis was performed in 25 g samples from each cheese pack as described [29]. After dilutions, the material was seeded in Baird-Parker Agar (BD, New Jersey, USA); up to five colonies of each morphology were selected, isolated, and preserved. In this first step, we obtained 75 colonies from the ten cheese packs. In a second step, colonies were seeded onto* S. saprophyticus* selective medium.

Water samples were obtained in two time points, December 2013 and March 2014 (single-day collection each), from beaches of Botafogo, Copacabana, Flamengo, Ipanema, and Leblon, in Rio de Janeiro city. Water was collected in collaboration with previous study [30] with modifications in the process of obtaining isolates. Briefly, in the first sampling section, water volumes inoculated in each Petri dish were 200 *μ*L without filtering and 10 mL, 20 mL, 50 mL, and 100 mL filtered in 0.22 *μ*m Millipore membranes (Merck, Darmstadt, Germany). In the second sampling section, after observing the high numbers of* S. saprophyticus* isolates obtained only from Leblon beach in the first section, only waters from this beach were sown in the five Petri dishes with different inoculum volumes. Waters from the other four beaches required just the 50 mL membrane filtration. Up to 16 colonies in each Petri dish were chosen and named according to the source and morphology.

All colonies were identified by biochemical tests [31] and matrix-assisted laser desorption ionization time of flight mass spectrometry (MALDI-TOF), using MALDI Biotyper 3.1 software (Bruker Daltonics) [32].* S. saprophyticus* American Type Culture Collection (ATCC) 15305 was the control test. Isolates identified as* S. saprophyticus* were stored in suspensions containing 10% (w/v) skimmed milk and 10% glycerol (v/v) in a freezer at −20°C.

### 2.2. Antimicrobial Susceptibility Test

The following antimicrobial agents (OXOID, Hampshire, England) were tested by disk diffusion method according to the Clinical and Laboratory Standards Institute (CLSI) [33, 34]: ciprofloxacin (5 *μ*g), clindamycin (2 *μ*g), erythromycin (15 *μ*g), gentamicin (10 *μ*g), levofloxacin (5 *μ*g), linezolid (30 *μ*g), nitrofurantoin (300 *μ*g), norfloxacin (10 *μ*g), penicillin (10 UI), and trimethoprim-sulfamethoxazole (1,25/23,75 *μ*g). Cefoxitin (30 *μ*g) disks predicted oxacillin resistance. Clindamycin inducible resistance was determined by the disk diffusion *D*-test. For antimicrobials with any isolate showing resistance in disk diffusion tests, minimum inhibitory concentrations (MIC) [33, 34] were determined by agar dilution.

### 2.3. Detection of Antimicrobial Resistance Genes

Resistance determinants investigated and primers used in this study are described in Table 1. We screened for the presence of genes related to antimicrobial agents showing resistance in disk diffusion tests, including genes described in* S. saprophyticus* or other* Staphylococcus* species. We confirmed one PCR product of each gene by sequencing and comparisons in GenBank with BLAST. Sequencing was additionally performed for some of the UTI isolates to verify the presence of mutations in* gyrA*,* par*C, and* pbp* (1 to 4) and the chromosomal dihydrofolate reductase. The* gyrA* and* par*C genes were characterized in the quinolone resistant isolate and four susceptible isolates randomly selected in order to observe specific amino acid substitutions [35, 36]. For the* pbp* genes, overlapping primer sequences were designed as to cover smaller fragments, using the* S. saprophyticus* ATCC 15305 as a template, to allow the determination of the entire gene sequences of the 32 UTI isolates.

### 2.4. Pulsed-Field Gel Electrophoresis

PFGE was performed with* Sma*I (20U, New England, Ipswich, USA), as described [37] with the following modifications: (i) bacterial suspensions were prepared in 500 *μ*L PIV buffer [NaCl 1 M and Tris HCl 10 mM (pH 7,6)] so that an aliquot of the 1 : 10 diluted suspension in distilled water exhibited an OD of 1.5 in a spectrophotometer with 600 nm wavelength; (ii) after solidification, blocks were transferred to lysing solution (Tris HCl 6 mM [pH 7,6], NaCl 1 M, EDTA 100 mM [pH 7,5], Brij 58 at 0.5%, sodium lauroyl sarcosinate at 0.5%, 0.5 mg/ml of lysozyme [Sigma], and 0.05 mg/ml lysostaphin [Sigma]) for 18–24 h at 37°C, under gentle agitation. Analysis of band profiles was performed with BioNumerics version 7.1 (Applied Maths, Kortrijk, Belgium) and dendrograms were built with Dice coefficient, 1.0% tolerance, using UPGMA. Clusters were named with capital letters (A–E) when belonging to the ITU genotypes. Clusters included isolates with ≥90% similarity or with one band difference.

### 2.5. Data Analysis

Data were analyzed with the free-access program OpenEpi version 3.03 [38]. Differences between categorical variables were analyzed by chi-square or Fisher's exact test. A *p* value ≤0.05 was defined as statistically significant.

## 3. Results and Discussion

### 3.1. Identification of* S. saprophyticus* from UTI, Pregnant Women's Microbiota, Minas Cheese, and Beach Water

The* S. saprophyticus* study collection included 32 isolates from UTI [11], 65 isolates from 23 (4% of 621) pregnant women, 30 isolates from waters of five beaches, and 13 isolates from two minas cheese packs (Table 2).

The finding of 4% pregnant women colonized with* S. saprophyticus* was below the colonization occurrence described in the three papers previously published, which reported (i) 11% of 123 women from a Kidney Clinic [18], (ii) 14% of 44 UTI cases [39], and (iii) 7% of 276 women in routine gynecological care [40]. However, our study population is higher (621 women), and the 95% confidence interval (CI) is the lowest among the others: CI of 0.02–0.05 from our study, (i) CI of 0.009–0.09, (ii) CI of 0.06–0.19, and (iii) CI of 0.03–0.08. Our finding is robust and suggests a reliable result. The study is refined by genotypic characterization of those isolates and reflects the presence of genes encoding antimicrobial resistance circulating in the human microbiota.

The 30 isolates identified as* S. saprophyticus* on the five beaches were obtained in both periods: 11 (37%) in December 2013 and 19 (63%) in March 2014. In recent years,* S. saprophyticus* has been described in aquatic environments taken as polluted, such as the marine environment in Lebanon [8] and river flood in Porto Alegre (Brazil) [7]. Here, we observed a high number (*p* ≤ 0.01) of* S. saprophyticus* isolates in Leblon beach, known to receive waters periodically from two fluvial channels carrying untreated domestic sewage. In periods of rain, the channels' floodgates are opened several times to decrease the water level, leading sewage into the sea. There were records of heavy rains during the days prior to sampling, indicating that this practice had recently happened; contamination of seawaters with untreated domestic sewage could explain the high numbers of* S. saprophyticus* in Leblon. In addition, pregnant women and beach water isolates shared similar resistance gene profiles, indicating a link between these sources.

MALDI-TOF MS confirmed identification of all 140 isolates with scores >2 in 84% and <2 in 16%. The scores were consistent with the biochemical tests used to identify* S. saprophyticus*, including values lower than the threshold of 2.0 for species identification (≥1.844), as described by others [32]. For coagulase-negatives, in general, this technique is capable of identifying species and subspecies with good performance [41].

The use of selective culture medium was instrumental. Mannitol Salt agar inhibited Gram-negative bacteria, and novobiocin improved the chances to obtain* S. saprophyticus* from cheese and the genital-anal microbiota; moreover, the addition of sodium azide [28] in this medium was essential to isolate* S. saprophyticus* from beach waters. Sodium azide shows bacteriostatic effect to most Gram-negative bacteria; Gram-positive bacteria are usually resistant to this compound [42].

### 3.2. Antimicrobial Resistance Phenotypes and Genes

Disk diffusion and MIC tests were congruent for susceptibility category.  Table 3 shows the analytical findings regarding phenotypic resistance and detection of antimicrobial resistance genes. Comparison of resistance genes present in isolates from the four collections showed a higher concentration of these genes in isolates from pregnant women and beach water (Table 4). This study is the first to report the resistance determinants* dfrA* and* dfrG* and* msr*(B) and* mph*(C) in* S. saprophyticus* obtained from humans.

Trimethoprim-sulfamethoxazole resistance, in 17% (*n* = 17) of isolates, correlated with the* dfrG* or* dfrA* genes, present in all resistant isolates, and only 10% (*n* = 10) of susceptible isolates (*p* < 0.0001). The sequence of the chromosomal* dfr* gene in all isolates was identical to that of trimethoprim susceptible ATCC 15305 strain, and the plasmid genes* dfrG* and* dfrA* shared 100% nucleotide identity with the respective variants described in other staphylococcal species including* S. aureus* (*dfrA*: ACSN01000071.1;* dfrG*: ABFA0100044.1),* S. epidermidis* (*dfrA*: NC_002976.3), and* S. pseudintermedius* (*dfrG*: NC_014925.1).

Although MLS_B_ antimicrobial agents are not recommended for UTI treatment [43], several genes that encode resistance to these agents were reported in* S. saprophyticus*. The most frequently described in this species is* erm*(C) [13, 14, 23, 44], almost universally present among our UTI isolates (*n* = 29/91%; Table 4). MLS_B_ resistance genes were present in resistant and susceptible isolates alike (Table 3). The* erm*(C) gene shared 100% nucleotide identity with those described in* S. aureus* (NC_007792.1) and* S. haemolyticus* (NC_007170.1). Both* msr*(A) and* msr*(B) genes shared 99% of nucleotide identity with* S. aureus* (CP002141.1) and* S. xylosus* (M81802.1), respectively. The* mph*(C) gene had 99% identity with those described in* S. aureus* (CP007177.1) and* S. epidermidis* (GQ900458.1).

In the more recent collections, a combination of genes replaced the* erm*(C) gene as a sole MLS_B_ resistance determinant, a difference certainly due to the time lag between strains' isolation periods (2005-2006 for UTI and 2011–2013 for other isolates) (Table 4). Concerning clindamycin resistance, only inducible phenotype was observed in 13% of isolates (*n* = 13). Resistant isolates had different combinations of* erm*(C),* msr*(A),* msr*(B),* mph*(C), and* lin*(A). The* lin*(A) gene shared 100% and 99% identity with the genes already described in* S. aureus* (EU350088.1) and* S. haemolyticus* (M14039.1), respectively.

*β*-Lactam resistance was rarely supported by molecular mechanism (Table 3). Oxacillin resistance was confirmed in only four isolates (4%) with the* mecA* gene, though 9% of isolates were resistant by disk diffusion and 93% by MIC determination. We observed 89 non-*mecA* isolates with oxacillin-resistant breakpoints, though with low MIC values (0.5 *μ*g/mL–2 *μ*g/mL). It has been suggested that the most appropriate breakpoints to cefoxitin disk diffusion and oxacillin MIC should be ≤19 mm and ≥16 *μ*g/mL, respectively, to indicate* mecA*-positive* S. saprophyticus* [45]. With these criteria, all 89* S. saprophyticus* isolates would be classified as susceptible. However, this MIC breakpoint [45] would be inappropriate for one of the* mecA*-positive isolates from beach waters, with MIC 1 *μ*g/ml, confirmed in triplicate. On the other hand, the suggested cefoxitin inhibition zone <19 mm would fit, since this* mecA*-positive isolate had a 12 mm halo.

Penicillin resistance by MIC correlated with the presence of* mecA* gene in only three isolates (Table 3). Other three penicillin-resistant isolates (MIC ≤ 1 *μ*g/mL) had no resistance determinant, including* blaZ* or PBP nucleotide sequence polymorphisms that distinguished resistant from susceptible isolates. By comparing PBP sequences of the 32 UTI isolates with those of* S. saprophyticus* ATCC 15305, we found only substitutions unrelated to phenotypic penicillin resistance in the PBP1 deduced amino acid sequence: V625M in 31 isolates and E731K in all 32 isolates. The contribution of the two substitutions in the deduced amino acid sequence of PBP1 to antimicrobial resistance is yet to be unraveled.

Two of the* mecA*-positive isolates were obtained from the microbiota of pregnant women (Table 4). The presence of this isolate may indicate risk of UTI by oxacillin-resistant* S. saprophyticus* to this group. Although* S. saprophyticus* is uncommon UTI agent in pregnant women [46], treatment of such infections in this population is performed with first-generation cephalosporins, such as cephalexin [47], a *β*-lactam without activity for* mecA*-carrying isolates. In addition, colonized pregnant women may represent a risk for transmission of resistant microorganisms to the newborn [48], as already shown to occur with resistant enterobacteria [49, 50].

Regarding other drugs, even though one isolate was intermediately resistant to norfloxacin, no* gyrA* and* parC* gene mutations were found (Table 3). It is possible that the result of the disk diffusion test was false-positive, since the MIC was susceptible and the corresponding mutations were not found with the resistance phenotype. In the case of the two isolates with intermediate resistance to gentamicin (Table 3), it was not possible to elucidate mechanisms of resistance through the aminoglycoside-modifying enzyme-encoding genes studied. This suggests that additional mechanisms of resistance may be involved in the expression of this phenotype for this microorganism.

### 3.3. PFGE Typing

We performed typing to investigate if multiple isolates from colonization in microbiota would be clonal and to compare our isolates from the multiple sources with the five PFGE clusters we detected previously.

**Figure 2:**
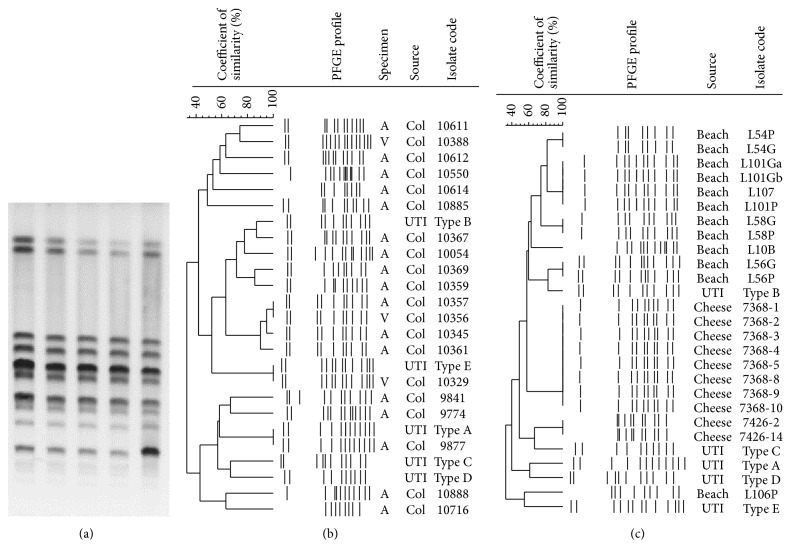
Pulsed-field gel electrophoresis profiles of the* Staphylococcus saprophyticus* isolates obtained from pregnant women, minas cheese, and beach waters of Rio de Janeiro. PFGE band patterns of (a) five isolates obtained from the microbiota of a single pregnant women; (b) comparison of the five uropathogenic PFGE clusters with one isolate per pregnant woman (*n* = 21) and (c) 10 isolates from minas cheese and 12 from beach waters. “A”: anal; “V”: vaginal.

From the total 23 pregnant women with* S. saprophyticus*, isolates from 21 were typeable. This collection included 43 isolates from 10 women with more than one isolate and one from each of 11 women with a single isolate (54 isolates in total). PFGE band profiles from a single individual were mostly clonal: six women had profiles 90%–100% similar (Figure 2(a)), and the other four were colonized with isolates 76%–100% similar (three band differences at most). Our strategy to type more than one colony per women to study the microbiota may be helpful for future epidemiological studies, as we demonstrated that* S. saprophyticus* isolates from the same person are usually clonal.

Comparison of PFGE profiles from different women (Figure 2(b)) showed that four of them shared profiles >90% similar, and comparison with the previously detected clones revealed that each of two women had isolates 100% similar with UTI type A and others with UTI type E. This result indicates that* S. saprophyticus* clones can stay successfully established in the environment and among people; UTI and pregnant women's microbiota PFGE types were obtained almost a decade apart. Similar results were previously observed in Germany but only among UTI isolates [51].

Of the 10 typeable isolates from cheese, only those from the same cheese pack were clonal (100% similar, Figure 2(c)). Our results indicate single original contamination in the cheese sample, but this may not be the rule, as another study in France showed polyclonal contamination in this same food [3]. It is possible that a large number of cheese packs from more brands would show a different clonal composition per cheese as the study in France. More studies regarding this issue are needed to support this food as a possible source of UTI. Despite the difference between the UTI and the cheese types in the present study, this food could be a brief point source of* S. saprophyticus* to humans.

We obtained 12 typeable isolates from beach water (all from Leblon beach). Only isolates from the same culture plate were clonal (100% similar, Figure 2(c)). Although we did not observe clusters with >90% similarity among beach waters and UTI isolates, one* S. saprophyticus* from beach and another from UTI (type B pattern) had similarity of 82% (two band differences). Further studies are needed to investigate a possible connection among beach water and human UTI isolates. Indeed, the association between ITU by* S. saprophyticus* and swimming activities was described long ago [52]. However, only a technique such as whole genome sequencing could propose the transmission direction of strains from food to humans.

PFGE performance was unsuccessful for 18* S. saprophyticus* isolates from beach waters. The reason for such failure may relate to bacterial survival strategy in saturated environment such as beach waters. Possible changes in* S. saprophyticus* cell wall could differentiate isolates in beach waters. Such differences were demonstrated in marine* Pseudomonas aeruginosa* [53]. Indeed, PFGE protocols are validated for clinical isolates and may be less suitable for environmental organisms.

Comparison among PFGE profiles of all isolates showed that the similarity detected in smaller groups of isolates remained (data not shown).

## 4. Conclusions

The presence of similar genotypic characteristics (high presence of resistance genes) in isolates from microbiota and beach waters indicates that* S. saprophyticus* may transit between both sites. Likewise, the two isolates with* mecA* gene and full resistance to oxacillin in beach waters alert to the possibility of transmission of this highly relevant resistance determinant to other humans. On the other hand, the finding of* S. saprophyticus* fully susceptible to antimicrobial agents in minas cheese suggests that these isolates represent microorganisms in their wild state in the environment.

Resistance to oxacillin and gentamicin was little related to genes screened in this study. Additional investigations are needed to assess the discrepancies observed here. The unconfirmed resistance to norfloxacin by MIC or mutations in QRDR regions indicated that the disk diffusion test was inaccurate.

The PFGE association of isolates from microbiota with 100% similarity, compared to two PFGE UTI cluster types obtained one decade apart, shows long-term persistence of uropathogenic types.

## Figures and Tables

**Table 1:** Primers used for screening of resistance determinants in *Staphylococcus saprophyticus*.

Antimicrobial	Gene	Sequence 5′-3′	Size (bp)	Ref.
Gentamicin	*aac(6*′*)-Ie+aph(2*′′)	CCAAGAGCAATAAGGGCATACC	347	[19]
		CACACTATCATAACCATCACCG		
	*ant(4*′*)-Ia*	CTGCTAAATCGGTAGAAGC	172	
		CAGACCAATCAACATGGCACC		
	*aph(3*′*)-IIIa*	CTGATCGAAAAATACCGCTGC	268	
		TCATACTCTTCCGAGCAAAGG		

Erythromycin	*erm*(A)	TATCTTATCGTTGAGAAGGGATT	533	[20]
		CTACACTTGGCTTAGGATGAAA		
	*erm*(B)	CTATCTGATTGTTGAAGAAGGATT	359	[21]
		GTTTACTCTTGGTTTAGGATGAAA		
	*erm*(C)	CTTGTTGATCACGATAATTTCC	190	[22]
		ATCTTTTAGCAAACCCGTATTC		
	*msr*(A)	GGCACAATAAGAGTGTTTAAAGG	940	[21]
		AAGTTATATCATGAATAGATTGTCCTGTT		
	*msr*(B)	TATGATATCCATAATAATTATCCAATC	595	
		AAGTTATATCATGAATAGATTGTCCTGTT		
	*lin*(A)	GGTGGCTGGGGGGTAGATGTATTAACTGG	323	
		GCTTCTTTTGAAATACATGGTATTTTTCGATC		
	*mph*(C)	GAGACTACCAAGAAGACCTGACG	722	[23]
		CATACGCCGATTCTCCTGAT		
	*mef*(A)	AGTATCATTAATCACTAGTGC	348	[24]
		TTCTTCTGGTACAAAAGTGG		

Oxacillin	*mecA*	GTGAAGATATACCAAGTGATT	147	[25]
		ATGCGCTATAGATTGAAAGGAT		

Penicillin^a^	*blaZ*	ATGTAATTCAAACAGTTCACATGCC	701	[26]
		ATAGGTTCAGATTGGCCCTTAGG		
	*pbp1A*			This study
	Fragment 1	GGCGAACATTCCACAGTGTTGACT	627	
		GGGTGTCAGTAAGGCGTTCAAACCA		
	Fragment 2	TCTCACCGTCACCAATAACGATTGG	680	
		TACAGGCGGATCGCTTGGG		
	Fragment 3	TCAGCTACTTGTGCGGTACCTGT	758	
		TTTGCAGTAGTGATGGACGCACA		
	Fragment 4	GCCGCCTGTAGCTTCACCGT	650	
		TGAAAGGTGCTATGGGCGTAGAGAA		
	Fragment 5	TGTGCGTCCATCACTACTGCAAA	788	
		GGGGCAGTCCTACTCATCCTTGGA		
	Fragment 6	GCCGACATATCAACAACGGTAGCCA	683	
		AGACCAAAACCGTAAAACGCAAGGT		
	*pbp2A*			This study
	Fragment 1	ACGTGCTGCACTTTGTTGGTTACT	775	
		TGCCGGAAACGATGCACCAA		
	Fragment 2	TTGGTGCATCGTTTCCGGCA	763	
		GCGGAATCTGCTTATCTTGCTGGT		
	Fragment 3	CGCGATCTTCAGATGAACGTTGGA	869	
		GCGAGGACCGCGTATGACGG		
	*pbp3A*			This study
	Fragment 1	GACGATTATGACGGCCTTTT	702	
		TGGATCAAGTGCAGAACCAG		
	Fragment 2	CCTGACAGTCAACGAAGCTG	769	
		AAGATCATCGCCACGTGAAC		
	Fragment 3	GCATTTTCGGTGACGTTTCT	714	
		CGCTCGTAATATCGGTTGGT		
	Fragment 4	TGATTGATGAGCCGCTTAAA	744	
		ATGGCTCAGGTACTGGTTGG		
	Fragment 5	GGCATTTAACGAAAAAGATGGA	410	
		TACGTTTACGCGCATGCTAA		
	*pbp4A*			This study
	Fragment 1	AATCCAGCGACAAACATCCCATTCA	835	
		AAGCGCAGCAGCATTACTAGAGTT		
	Fragment 2	GGCGCCATTAATGTTTCGCAAACA	718	
		CGCCGGCGCCCATGATAACT		
	Fragment 3	GGTGCCGATATGAGCCTAGAAGGT	859	
		TGGGCCATGATTGGGAAGGCG		

Trimethoprim	*dfrD*	CCCTGCTATTAAAGCACC	606	[27]
		CATGACCAGATAACTC		
	*dfrG*	TGCTGCGATGGATAAGAA	405	[26]
		TGGGCAAATACCTCATTCC		
	*dfrK*	CAAGAGATAAGGGGTTCAGC	229	
		ACAGATACTTCGTTCCACTC		
	*dfrS1*(*dfrA*)	CACTTGTAATGGCACGGAAA	270	
		CGAATGTGTATGGTGGAAAG		
	*dfr* ^b^	AATGGACATCGGTTGGGTTGCCT	484	This study
		CGCACCGGATTCAAATGTCTCGC		

Norfloxacin	*gyrA*	CGAGTGAGATGCGCGAGTCATTCTT	731	This study
		ACGTTGACGACCGCCACCAC		
	*parC*	ACGTTCGTGATGGGCTCAAACCT	797	
		ACGTAATCCAGTACGGTCTGTCTCA		

Ref.: reference. ^a^PCR primers were designed to target smaller *pbp* 1-4 gene fragments and allow for the respective whole gene sequence amplification; ^b^chromosomal *dihydrofolate reductase*.

**Table 2:** Distribution of *Staphylococcus saprophyticus* isolates obtained from pregnant women's microbiota, beaches, and minas cheese in Rio de Janeiro city.

Sample source	Number and % of *S. saprophyticus* isolates
Microbiota (23 positive women)	
Vaginal (3)	11, 17% (1–5 per woman)
Anal (16)	27, 41.5% (1–4 per woman)
Both (4)	27, 41.5% (3–9 per woman)
Total	65

Beach water (10 samples)	
Botafogo (2)	6, 20% (1–5 per sample)
Copacabana (2)	1, 3% (0-1 per sample)
Flamengo (2)	1, 3%
Ipanema (2)	1, 3%
Leblon (2)	21^*∗*^, 70%
Total	30

Minas cheese (10 cheeses)	
Brand A (4)	9, 69.2% (0–9 per cheese)
Brand B (3)	4, 30.7% (0–4 per cheese)
Brand C (3)	0
Total	13, 100%

Total of *S. saprophyticus* isolates	108

^*∗*^The comparison among *S. saprophyticus* obtained from each beach by Fisher's exact test showed that Leblon had significantly higher numbers of isolates than Botafogo, Copacabana, Flamengo, and Ipanema (*p* ≤ 0.01).

**Table 3:** Antimicrobial susceptibility and resistance determinants in 98 *Staphylococcus saprophyticus *isolates.

Antimicrobial agent	Disk diffusion halo (mm)/interpretation	MIC (*μ*g/mL)/interpretation	Resistance determinant	
			Gene	Number (%) of isolates
Sulfamethoxazole-trimethoprim	6–12/R	4–64/R	*dfrG*	15 (15.3)
	6/R	32/R	*dfrA*	1 (1)
	11/I	8/R	*dfrG*	1 (1)
	18–35/S	0.125–4/S	*dfrG*	10 (10.2)
	29–37/S	0.125–2/S	None	71 (72.4)

MLS_B_				
Erythromycin	6/R	4–≥64/R	*erm*(C)	7 (7.1)
	6/R	32–>64/R	*erm*(C), *msr*(B)	2 (2)
	6/R	>64/R	*erm*(C), *mph*(C)	2 (2)
	6/R	≥64/R	*msr*(A), *msr*(B)	1 (1)
	6/R	≥64/R	*erm*(C), *msr*(A), *msr*(B)	9 (9.1)
	6–10/R	32–≥64/R	*erm*(C), *msr*(A), *msr*(B), *mph*(C)	10 (10.2)
	19/I	0.125/S	*erm*(C)	1 (1)
	22–33/S	0.125–1/S	*erm*(C)	44 (44.8)
	30/S	1/S	*msr*(A)	1 (1)
	28–32/S	0.25–1/S	*erm*(C), *msr*(A)	7 (7.1)
	28–34/S	1/S	*erm*(C), *msr*(B)	3 (3)
	27–30/S	1/S	*erm*(C), *mph*(C)	3 (3)
	28–30/S	1/S	*erm*(C), *msr*(A), *msr*(B)	1 (1)
	26–32/S	0.125–1/S	None	7 (7.1)

Clindamycin	30–33/IR^a^	0.125–2/S	*erm*(C)	6 (6.1)
	34/IR^a^	0.25/S	*erm*(C), *msr*(B)	1 (1)
	28–30/IR^a^	0.25/S	*erm*(C), *msr*(A), *msr*(B)	2 (2)
	28–32/IR^a^	0.25/S	*erm*(C), *msr*(A), *msr*(B), *mph*(C)	4 (4)
	25/S	0.25/S	*lin*(A),* erm*(C), *msr*(A), *msr*(B), *mph*(C)	1 (1)
	25/S	0.25/S	*lin*(A),* erm*(C)	1 (1)
	25–35/S	0.125–0.25/S	None	83 (84.6)

Oxacillin	6–9/R	32–>64/R	*mecA*	3 (3)
	12/R	1/R	*mecA*	1 (1)
	20–22/R	0.5–1/R	None	5 (5.1)
	23–35/S	0.5–2/R	None	84 (85.7)
	29–44/S	0.125–0.25/S	None	5 (5.1)

Penicillin	6/R	8–16/R	*mecA* ^b^	3 (3)
	15/R	2/R	None	1 (1)
	26–28/R	0.25–1/R	None	2 (2)
	22/R	0.125/S	*mecA* ^b^	1 (1)
	17–27/R	0.125/S	None	9 (9.1)
	30–45/S	≤0.125/S	None	82 (83.6)

Gentamicin	14/I	8/I	None	1 (1)
	15/S	8/I	None	1 (1)
	15–37/S	0.125/S	None	96 (97.9)

Norfloxacin	15/I	0.125/S	None	1 (1)
	17–33/S	0.125/S	None	97 (98.9)

The 98 isolates in this table were selected from the 140 obtained to include only one isolate per patient. MLS_B_: *macrolide-lincosamide-streptogramin* B; R: resistant; I: intermediate resistance; S: susceptible; IR: inducible resistance; MIC: minimum inhibitory concentration. ^a^All inducible resistance detected in *D*-test. ^b^Same isolates resistant to oxacillin.

**Table 4:** Distribution of the antimicrobial resistance genes by sample source among the 98 *Staphylococcus saprophyticus* isolates.

Gene	Number and % in each collection				
	UTI/2005-2006 *N* = 32	Pregnant women/2011–2013 *N* = 23	Beach water/2013-2014 *N* = 30	Cheese/2011 *N* = 13	Total *N* = 98
MLS_B_					
*ermC*	24 (75)	6 (26)	10 (33.3)	11 (84.6)	78 (79.5)
*msrA*	0 (0)	0 (0)	1 (3.3)	0	34 (34.6)
*msrB*	0 (0)	0 (0)	0 (0)	0	26 (26.5)
*mphC*	0 (0)	0 (0)	0 (0)	0	18 (18.3)
*ermC*,* msrA*	0	4 (17.3)	3 (10)	0	7 (7.1)
*ermC*,* msrB*	0	2 (8.6)	1 (3.3)	0	3 (3)
*ermC*,* linA*	0	0	1 (3.3)	0	1 (1)
*ermC*,* mphC*	0	2 (8.6)	3 (10)	0	5 (5.1)
*msrA*,* msrB*	0	0	1 (3.3)	0	1 (1)
*ermC*,* msrA*,* msrB*	5 (16)	2 (8.6)	3 (10)	0	10 (10.2)
*ermC*,* msrA*,* mphC*	0	2 (8.6)	0	0	2 (2)
*ermC*,* msrA*,* msrB*,* mphC *	0	4 (17.3)	5 (16.6)	0	10 (10.2)
*ermC*,* msrA*,* msrB*,* mphC*,* linA*	0	0	1 (3.3)	0	1 (1)
>1 gene^a^	5 (15.6)	16 (69.5)	20 (20.4)	0	41 (41.8)

Other					
*dfrG*	16 (50)	7 (30.4)	3 (10)	0	26 (26.5)
*dfrA*	1 (3.1)	0 (0)	0 (0)	0	1 (1)
*mecA*	0 (0)	2 (8.6)	2 (6.6)	0	4 (4)

MLS_B_: macrolide-lincosamide-streptogramin B. ^a^*p* < 0.001 for comparison of >1 gene present among isolates from UTI or cheese and pregnant women and from UTI or cheese and beach water.
